# LINC00514 upregulates CCDC71L to promote cell proliferation, migration and invasion in triple‐negative breast cancer by sponging miR-6504-5p and miR-3139

**DOI:** 10.1186/s12935-021-01875-2

**Published:** 2021-03-23

**Authors:** Xiao Luo, Hui Wang

**Affiliations:** 1 Department of Breast Surgery, China-Japan Union Hospital of Jilin University, Changchun, 130033 Jilin China; 2 Department of Ultrasound, China-Japan Union Hospital of Jilin University, Changchun, 130033 Jilin China

**Keywords:** LINC00514, miR-6504-5p, miR-3139, CCDC71L, TNBC

## Abstract

**Background:**

Long noncoding RNAs (lncRNAs) have recently identified as essential gene modulators in numerous cancers. Previous studies have confirmed the oncogenic role of long intergenic nonprotein-coding RNA 00514 (LINC00514) in some cancers. Nevertheless, its biological function and mechanism remain unclear in triple-negative breast cancer (TNBC).

**Methods:**

Herein, we detected LINC00514 expression level in TNBC tissues and cells using RT-qPCR. The function of LINC00514 in TNBC cellular activities was assessed by colony formation, EdU, wound healing, transwell assays and flow cytometry analysis.

**Results:**

The binding between miR-6504-5p/miR-3139 and LINC00514/CCDC71L was validated by luciferase reporter assay. The results indicated that LINC00514 expression was upregulated in TNBC tissues and cells. Furthermore, it was manifested that silenced LINC00514 restrained cell proliferative, migratory and invasive abilities and promoted cell apoptosis. In mechanism, LINC00514 was revealed to sequester miR-6504-5p and miR-3139 in TNBC cells. Furthermore, the low level of miR-6504-5p and miR-3139 was identified in TNBC tissues and cells. Overexpression of miR-6504-5p or miR-3139 inhibited cell growth and migration in TNBC. CCDC71L was recognized as a common downstream gene of miR-6504-5p and miR-3139. Rescue assay verified that overexpressed CCDC71L countervailed the anti-tumor influence of LINC00514 knockdown on TNBC cell proliferation, migration, invasion and apoptosis.

**Conclusions:**

LINC00514 promote cell proliferation, migration and invasion in triple-negative breast cancer by targeting the miR-6504-5p/miR-3139/CCDC71L axis in TNBC.

**Supplementary Information:**

The online version contains supplementary material available at 10.1186/s12935-021-01875-2.

## Introduction

Breast cancer (BC) has been commonly acknowledged as a cancer prevalent among women globally [1]. It has various subtypes with multiple clinical outcomes and diverse biological behaviors because of the heterogeneous property [2]. Triple-negative breast cancer (TNBC) is an aggressive subtype losing Her2 amplification, progesterone receptor and estrogen receptor, and accounts for about 10–25% of total BC cases [3, 4]. Additionally, TNBC patients present worse clinical outcome, higher incidence and higher risk of distant metastasis [5, 6]. Thus, it is quite urgent to probe the novel biomarkers and potential molecular mechanisms underlying TNBC progression.

As a group of noncoding RNAs, long noncoding RNA (lncRNA) lacks protein translation abilities and comprises over 200 nucleotides [7]. Abnormally expressed lncRNAs are commonly discovered in multiple cancer types and their dysregulation is widely reported in numerous cellular activities, such as cancer initiation, apoptosis, migration and metastasis [8]. LncRNA SNHG1 is highly expressed in non-small-cell lung cancer and aggravates proliferative and invasive capacities of cells [9]. Downregulation of BLACAT1 suppresses cell proliferation and arrests cell cycle at G0/G1 phase in cervical cancer [10]. Besides, some lncRNAs have been recognized to play critical roles in TNBC [11, 12]. For example, lncRNA MIR100HG is identified as oncogenic gene that promotes cell proliferation in TNBC [13]. LncRNA linc-ZNF469-3 is upregulated and enhanced lung metastasis in TNBC [14]. Therefore, exploration for the new biomarkers from lncRNAs may provide a promising choice for the therapy of TNBC.

LncRNAs have been revealed to act as biological regulators through a variety of mechanisms [15], such as regulating alternative splicing [16], modifying chromosome [17], and sponging microRNA (miRNA) [18]. Long intergenic nonprotein-coding RNA 00514 (LINC00514) is a newly identified lncRNA, which exerts functions in cancers. Nowadays, great attention has been attracted on LINC00514 due to its critical role in the malignancy of papillary thyroid cancer [19] and the progression of osteosarcoma [20]. Nevertheless, the detail function and regulatory mechanism of LINC00514 in TNBC remain unclear. In this study, the high expression of LINC00514 was identified in TNBC tissues and cells, and LINC00514 played oncogenic role in TNBC via miR-6504-5p/miR-3139/CCDC71L axis.

## Materials and methods

### Tissue samples

TNBC samples and corresponding adjacent samples were collected from fifty-two TNBC patients at China-Japan Union Hospital of Jilin University (Jilin, China). Informed consents were signed by each patient. Prior to operation, no patients underwent radiotherapy or chemotherapy. After collecting, the samples were instantly preserved at − 80 °C in liquid nitrogen for further use. The use of tissues has obtained the approval from the Ethical Review Board of China-Japan Union Hospital of Jilin University (Jilin, China).

### Cell lines

Human normal breast epithelial cell (MCF-10 A), human TNBC cell lines (MDA-MB-468, HCC1937, MDA-MB-436, MDA-MB-231) and HEK293T cell line were all purchased from ATCC, and maintained in an incubator containing 5% CO_2_ at 37 °C. DMEM medium (Thermo Fisher Scientific, CA, USA) with 10% FBS and 1% Pen/Strep solution was used for cell culture.

### Cell transfection

The specific shRNAs targeting LINC00514 (sh-LINC00514#1/2) were obtained from GenePharma Company to silence LINC00514 in TNBC cells using Lipofectamine 2000 (Invitrogen, Carlsbad, CA, USA) with sh-NC as the negative control. The pcDNA3.1/LINC00514, pcDNA3.1/CCDC71L and empty NC vector, and miR-6504-5p/miR-3139 mimics/inhibitor and NC mimics/inhibitor, were all provided by GenePharma Company. Cells were reaped after 48 h.

### RT-qPCR

The extraction of total RNAs from tissues and cells was conducted by TRIzol reagent (Invitrogen) with a RecoverAll™ Total Nucleic Acid Isolation kit (Ambion). Reverse transcription reactions were performed using a Prime Script™ RT reagent kit (Takara, Dalian, China). Later, RT-qPCR was performed with SYBR Premix Ex Taq (Takara Bio, Shiga, Japan) on StepOnePlus System (Applied Biosystems). Based on the 2^−ΔΔCT^ method, gene expressions were calculated with normalization to GAPDH or U6.

### Colony formation assay

Transfected TNBC cells were seeded to 6-well plates, and each well was filled with 5 × 10^3^ cells. After 2 weeks, cells were fixed by 5% paraformaldehyde, and then 0.1% crystal violet solution was used for staining. Finally, the number of colonies (more than 50 cells) was counted and recorded.

### EdU assay

For the measurement of cell proliferation, Edu assay kit (RiboBio, China) was used. In brief, TNBC cells in each group with treatment of EdU were stained by DAPI. Under fluorescence microscope (Nikon, Japan), visualized images of EdU-positive cells were obtained.

### Flow cytometry analysis

TNBC cells were seeded in 6-well plates and then rinsed in PBS. Later, 1 µL of PI (Invitrogen) and 2.5 µL Annexin V conjugated with FITC were added into binding buffer. Subsequently, the binding buffer was used to resuspend the cells after trypsinization. After 15 min, apoptotic TNBC cells were measured by flow cytometry (BD Biosciences).

### Western blot analysis

Proteins in TNBC cells were extracted using RIPA buffer (Thermo Fisher Scientific), and then protein concentration was confirmed by BCA-kit (Beyotime, Shanghai, China). Separated by SDS-PAGE, proteins were transferred to PVDF membranes. Then, the membranes were blocked in 5% skim milk and incubated with primary antibodies (Abcam, Cambridge, MA) overnight at 4 °C. Washed by 0.1% TBST in triple, the membranes were incubated with secondary antibody at 37 °C for an hour. The internal control was GAPDH. The results were analyzed and visualized by ECL detection reagent (GE Healthcare, Chicago, IL).

### Wound healing assay

To make cells adhere, TNBC cells were cultured in 96-well plates all night with each well filled by 5 × 10^4^ cells. Later, sterile pipette tip was used to scratch the wounds. After 24 h, wounds were imaged following washing with PBS.

### Transwell invasion assay

Transfected TNBC cells were reaped and placed into the upper transwell chamber (8 μm pores) coated with Matrigel (BD Biosciences). The serum-free medium was placed in the upper chambers, while the lower chamber was supplemented with medium containing 10% FBS. Incubated for 24 h, cells were fixed and stained. Then, an inverted microscope was used to count invasive cells.

### Subcellular fractionation assay

PARIS Kit (Invitrogen) was used to isolated nuclear and cytoplasmic RNA in TNBC cells. Then, the extraction of subcellular fractions was carried out. Later, the fractions were subjected to RT-qPCR with normalization to GAPDH (cytoplasm control) or U6 (nucleus control).

### RNA pull down assay

LINC00514 and NC-lncRNA labeled with biotin were transfected into MDA-MB-468 and HCC1937 cells. The lysates of TNBC cells were used to incubate with streptavidin magnetic beads for 4 h at 4 °C. Subsequently, precooled lysis buffer and salt buffer was used to rinse the beads. With the extraction of pull-down RNAs, the levels of miRNAs binding to LINC00514 were detected.

### Luciferase reporter assay

LINC00514-WT/Mut and CCDC71L-WT/Mut vectors were separately constructed by cloning wild type (WT) and mutant (Mut) miR-6504-5p or miR-3139 binding site in LINC00514 sequence or CCDC71L 3′-UTR to pmirGLO (Promega) vectors. Then, above luciferase vectors were transfected with miR-6504-5p mimics, miR-3139 mimics or NC mimics into TNBC cells for 48 h. Finally, luciferase activity was examined with Dual Luciferase Assay System (Promega).

### Statistical analysis

The statistical analysis was conducted using GraphPad Prism 6 (GraphPad). Data comparison between or over two groups were statistically analyzed by Student’s t test or one-way ANOVA. Three biological repeats were included in all experimental procedures with results presenting as the mean ± SD. The analysis of correlation between genes was conducted by Spearman’s correlation analysis. The value of *p* less than 0.05 was considered as cut-off value.

## Results

### LINC00514 was an upregulated lncRNA in TNBC

**Fig. 1:**
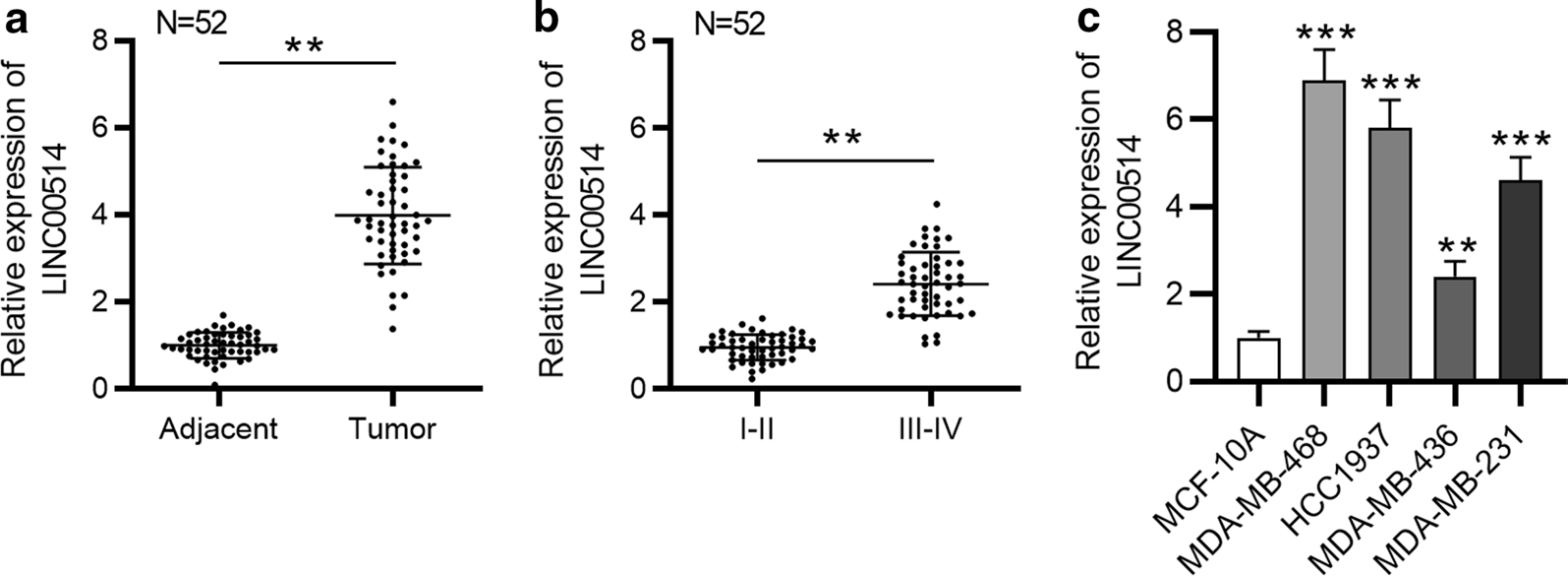
LINC00514 expression in TNBC tissues and cells. **a** RT-qPCR analysis of LINC00514 expression in TNBC tissues, with adjacent nontumor tissues as control. **b** Correlation of LINC00514 expression with clinical stage. **c** LINC00514 expression in TNBC cell lines was confirmed by RT-qPCR. **p < 0.01, ***p < 0.001

First, we analyzed LINC00514 expression profile in TNBC tissues and cell lines by RT-qPCR. Compared with corresponding adjacent tissues, LINC00514 expression was significantly upregulated in TNBC tissues (Fig. 1a). Importantly, LINC00514 expression was higher in patients at advanced stage (III-IV stage) than that in early stage (I-II stage) (Fig. 1b). In addition, upregulation of LINC00514 was found in TNBC cell lines with comparison of MCF-10 A cell line (Fig. 1c). Taken together, LINC00514 was highly expressed in TNBC tissues and cell lines.

### LINC00514 promoted TNBC cell proliferation, migration, invasion and inhibited cell apoptosis

**Fig. 2:**
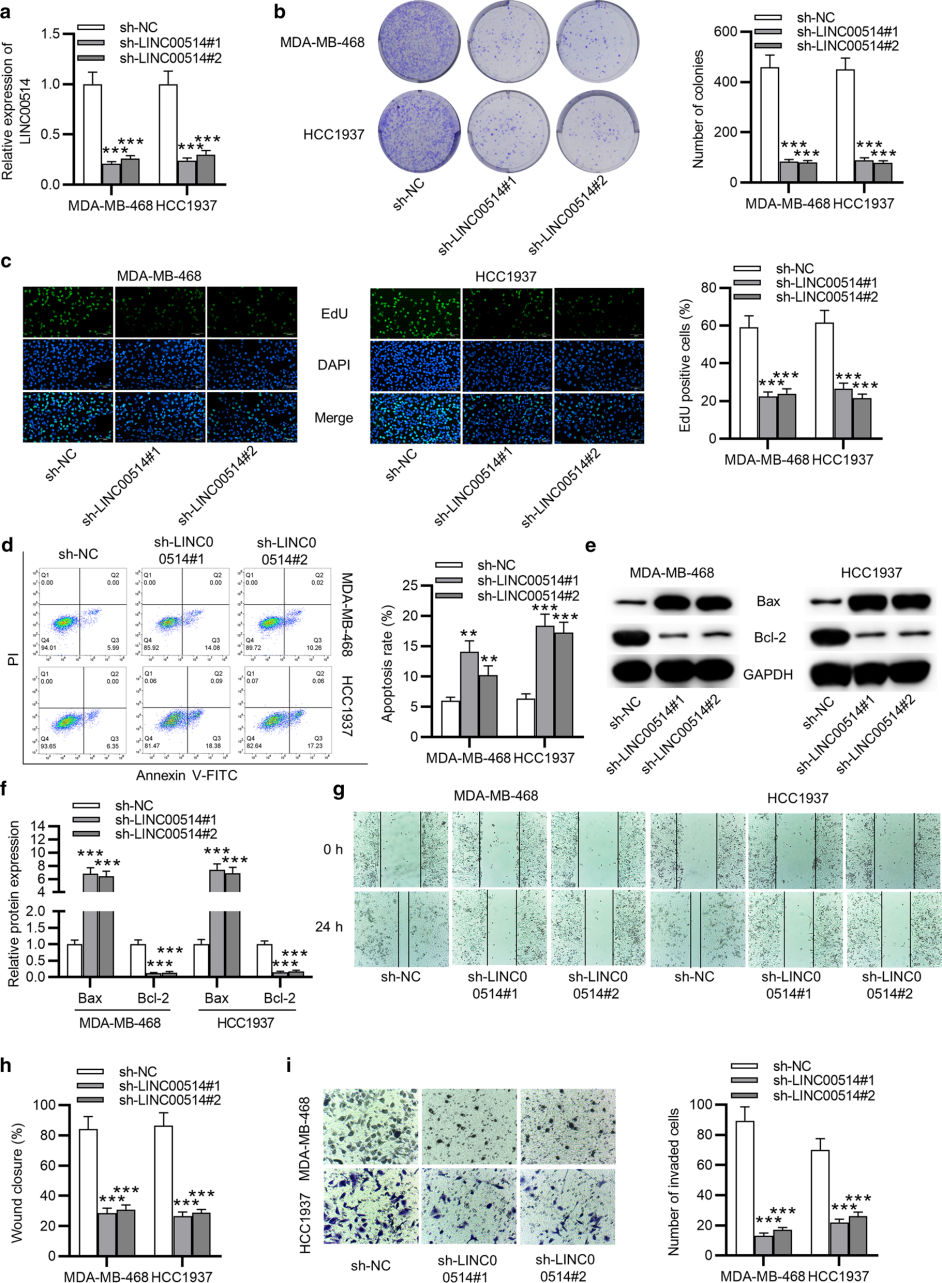
LINC00514 knockdown played inhibitory role in TNBC cell growth and migration. **a** Transfection efficiency of sh-LINC00514 in MDA-MB-468 and HCC1937 cells was evaluated by RT-qPCR. **b**, **c** Colony formation assay and EdU assay were carried out to assess cell (MDA-MB-468, HCC1937) proliferative ability with LINC00514 silencing. **d** Apoptosis of sh-LINC00514 transfected TNBC cells was detected by flow cytometry analysis. **e**, **f** Impact of LINC00514 deficiency in levels of apoptosis-associated proteins was evaluated with western blot. **g**, **h** Wound healing assay verified migratory ability of LINC00514-depleted TNBC cells. **i** Transwell assay was performed detecting cell invasive ability under LINC00514 knockdown. **p < 0.01, ***p < 0.001

Considering aberrant LINC00514 expression in TNBC, first we accessed the effect of LINC00514 overexpression in TNBC on MCF10A cells. The result of colony formation assay showed that LINC00514 overexpression had no significant influence on the proliferation of MCF10A cells (Additional file 1: Fig. S1 A). We then explored its biological function in TNBC via conducting loss-of-function assays. MDA-MB-468 and HCC1937 cells, which showed higher LINC00514 expression, was used for the investigation. LINC00514 expression was stably silenced by transfecting sh-LINC00514#1/2 in MDA-MB-468 and HCC1937 cells (Fig. 2a). Then, colony formation assay showed that cell proliferation was repressed by LINC00514 downregulation (Fig. 2b). Consistently, the same result was observed in EdU assay (Fig. 2c). However, the apoptosis rate was elevated in LINC00514-silenced TNBC cells through flow cytometry analysis (Fig. 2d). Western blot manifested the upregulation of Bax protein level and the downregulation of Bcl-2 protein level in sh-LINC00514 group (Fig. 2e, f). According to wound healing assay, cell migration was suppressed by silencing LINC00514 (Fig. 2g, h). Likewise, LINC00514 knockdown inhibited cell invasion via the result of transwell assay (Fig. 2i). Overall, LINC00514 accelerated cell proliferation, migration and invasion, and inhibited cell apoptosis in TNBC.

### LINC00514 sponged miR-6504-5p and miR-3139 in TNBC

**Fig. 3:**
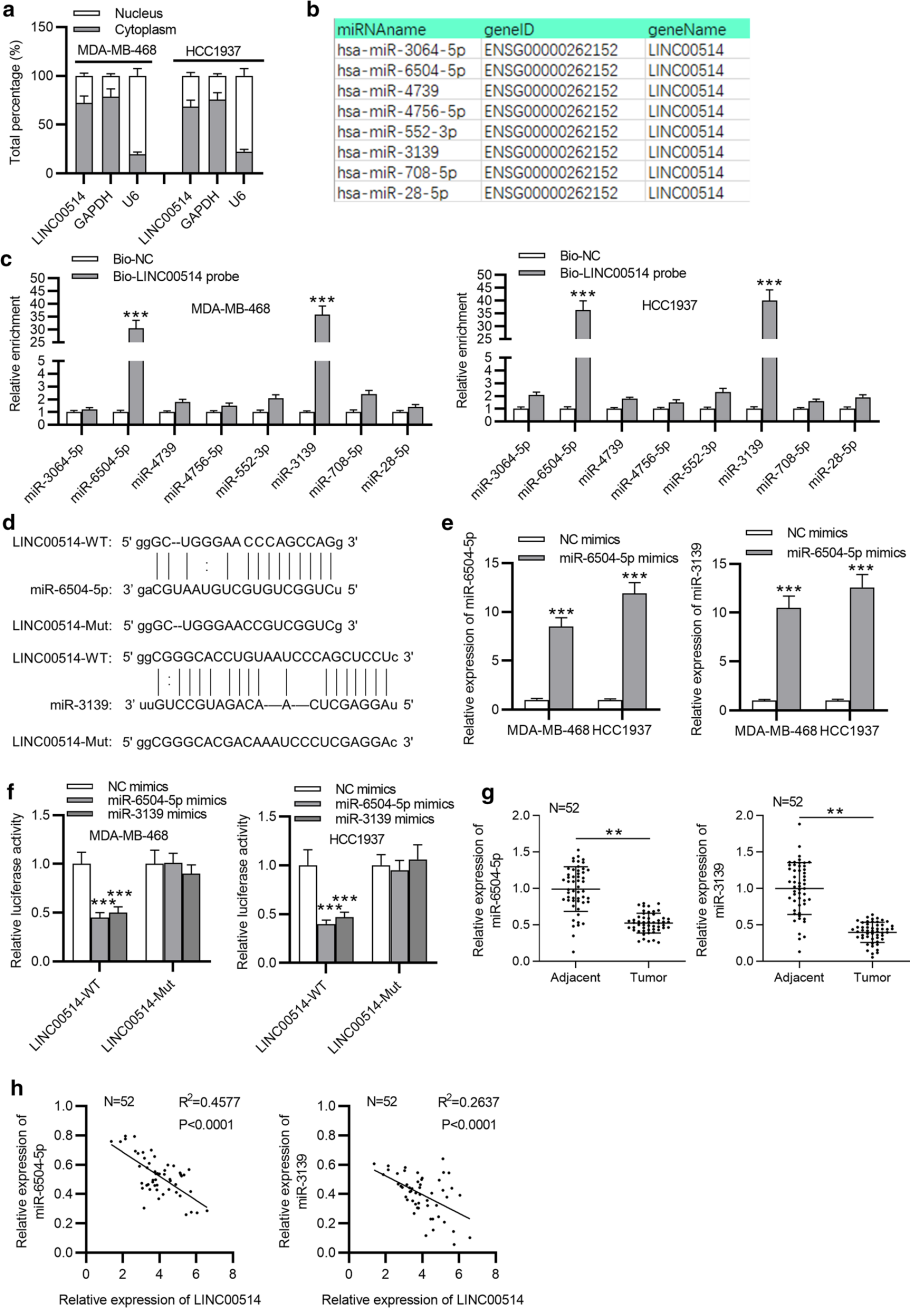
MiR-6504-5p and miR-3139 were the downstream miRNAs for LINC00514. **a** Subcellular fractionation assay determined LINC00514 location in TNBC cells. **b** StarBase predicted miRNAs binding to LINC00514. **c** RNA pull-down assay was utilized for validating the binding of LINC00514 to potential miRNAs. **d** The miR-6504-5p/miR-3139 binding sites for LINC00514 were predicted by starBase and their matched mutated sites were designed. **e** RT-qPCR analysis of miR-6504-5p/miR-3139 expression in MDA-MB-468 and HCC1937 cells transfected with miR-6504-5p/miR-3139 mimics. **f** Luciferase reporter LINC00514-WT/Mut was built to verify the binding of LINC00514 to miR-6504-5p/miR-3139. **g** RT-qPCR analysis of miR-6504-5p expression or miR-3139 expression in TNBC tissues and paired adjacent tissues. **h** Correlation analysis of LINC00514 expression and miR-6504-5p/miR-3139 expression in TNBC tissues. **p < 0.01, ***p < 0.001

Thereafter, we investigated the molecular mechanism of LINC00514 in TNBC. Firstly, we conducted subcellular fractionation assay, and the result revealed that LINC00514 was primarily distributed in the cytoplasm of TNBC cells (Fig. 3a). Increasing evidence suggests that lncRNA acts as a competing endogenous RNA (ceRNA) in various cancers [21, 22]. Therefore, we hypothesized that LINC00514 might act as a ceRNA through sequestering some specific miRNAs to mediate TNBC progression. Then, we searched the potential miRNAs for LINC00514 by starBase (http://starbase.sysu.edu.cn/). As a result, 8 miRNAs were screened out (Fig. 3b). Based on the RNA pull-down assay, miR-6504-5p and miR-3139 were observed to be pulled down by LINC00514 biotin probe compared with Bio-NC group (Fig. 3c). Thus, we speculated that miR-6504-5p and miR-3139 served as potential miRNAs for LINC00514 in TNBC. As shown in Fig. 3d, binding sites of miR-6504-5p or miR-3139 in LINC00514 sequence (LINC00514-WT) and the mutant site (LINC00514-Mut) were presented. Through RT-qPCR, we found miR-6504-5p or miR-3139 expression was significantly increased by respectively transfecting miR-6504-5p mimics or miR-3139 mimics (Fig. 3e). Luciferase reporter assay demonstrated that the luciferase activity of LINC00514-WT reporter was decreased by the overexpression of miR-6504-5p or miR-3139, while no significant change was found in that of LINC00514-Mut reporter (Fig. 3f). This revealed that both miR-6504-5p and miR-3139 interacted with LINC00514. Moreover, miR-6504-5p and miR-3139 were validated to be downregulated in TNBC tissues (Fig. 3g). Importantly, the expression of LINC00514 was negatively associated with that of miR-6504-5p or miR-3139 (Fig. 3h). Data above confirmed that LINC00514 served as sponge of miR-6504-5p and miR-3139 in TNBC.

### MiR-6504-5p and miR-3139 were lowly expressed in TNBC and inhibited cell proliferation, migration, invasion and promoted cell apoptosis

**Fig. 4:**
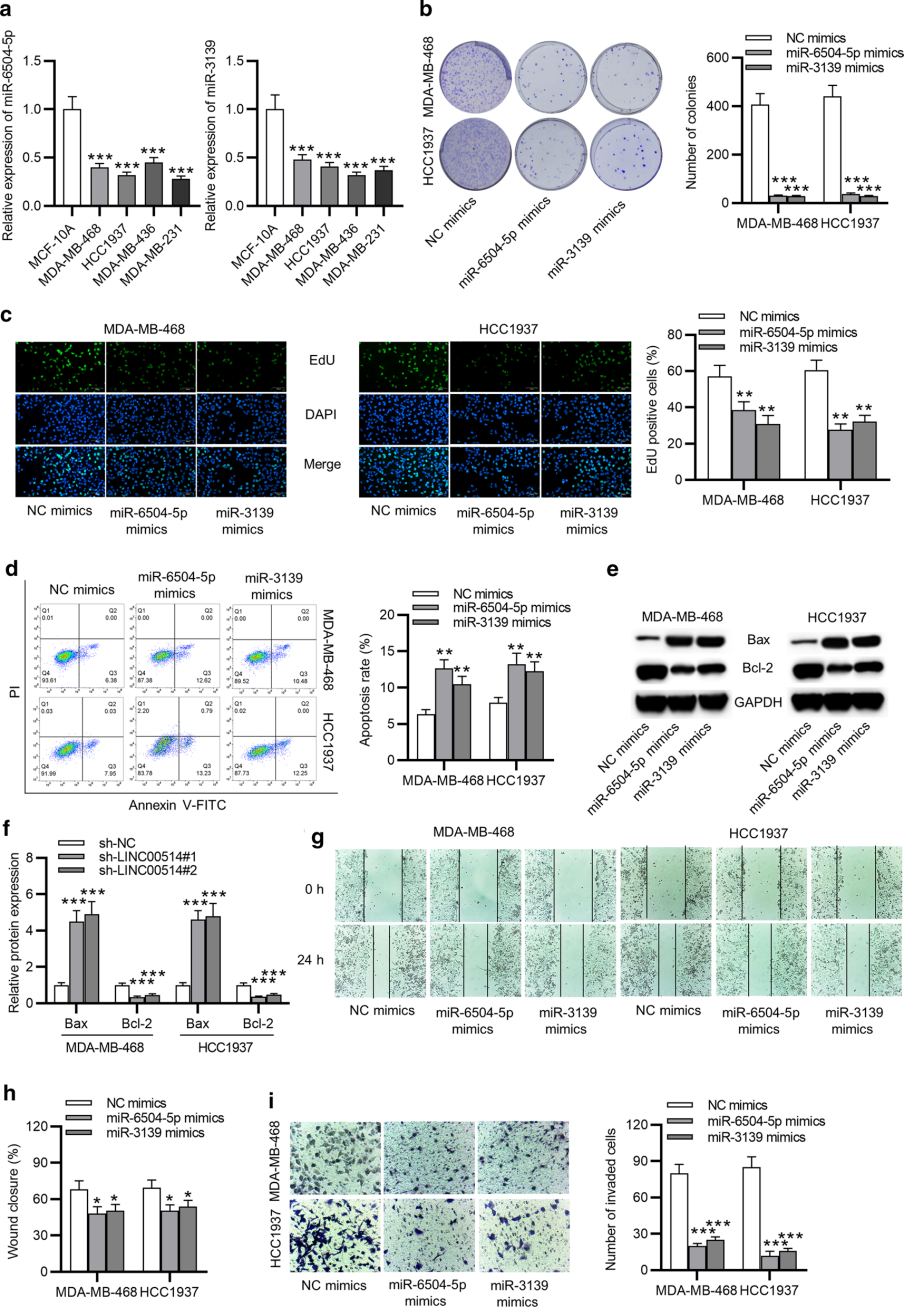
The inhibitive effect of overexpressed miR-6504-5p or miR-3139 on TNBC cell proliferation, migration and invasion. **a** RT-qPCR of miR-6504-5p and miR-3139 expression in TNBC cells and MCF-10 A cells. **b**, **c** The proliferation in TNBC cells with miR-6504-5p/miR-3139 mimics transfection was evaluated through colony formation and EdU assays. **d** Flow cytometry analysis demonstrated the function of upregulated miR-6504-5p and miR-3139 on cell apoptosis. **e**, **f** Bax and Bcl-2 protein levels in TNBC cells with transfection of miR-6504-5p/miR-3139 mimics or NC mimics. **g**, **h** Impact of upregulated miR-6504-5p/miR-3139 on TNBC cell migratory ability. **i** Transwell assay evaluated the invasion in miR-6504-5p/miR-3139 overexpressed cells. *p < 0.05, **p < 0.01, ***p < 0.001

Subsequently, we explored the expression pattern and biological functions of miR-6504-5p and miR-3139 in TNBC cells. Results of RT-qPCR depicted that miR-6504-5p and miR-3139 both expressed at a low level in TNBC cell lines (Fig. 4a). Subsequently, some functional assays were carried out to identify functional role of miR-6504-5p and miR-3139 in TNBC cell growth and migration. Based on results of colony formation and EdU assays, we found miR-6504-5p mimics and miR-3139 mimics inhibited cell proliferation (Fig. 4b, c). As shown in Fig. 4d, the apoptosis of TNBC cells was enhanced by overexpressed miR-6504-5p or miR-3139. In addition, levels of apoptosis-relevant proteins (Bax and Bl-2) in cells with transfection of miR-6504-5p mimics or miR-3139 mimics were tested. The results indicated that upregulation of miR-6504-5p or miR-3139 increased Bax protein level and reduced Bcl-2 protein level (Fig. 4e, f). Through wound healing assay, overexpression of miR-6504-5p or miR-3139 remarkably suppressed cell migratory capability (Fig. 4g, h). Furthermore, transwell assay confirmed the inhibitive role of miR-6504-5p or miR-3139 overexpression in TNBC cell invasion (Fig. 4i). Conclusively, miR-6504-5p and miR-3139 retarded cell growth and migration in TNBC.

### CCDC71L was a common target of miR-6504-5p and miR-3139

**Fig. 5:**
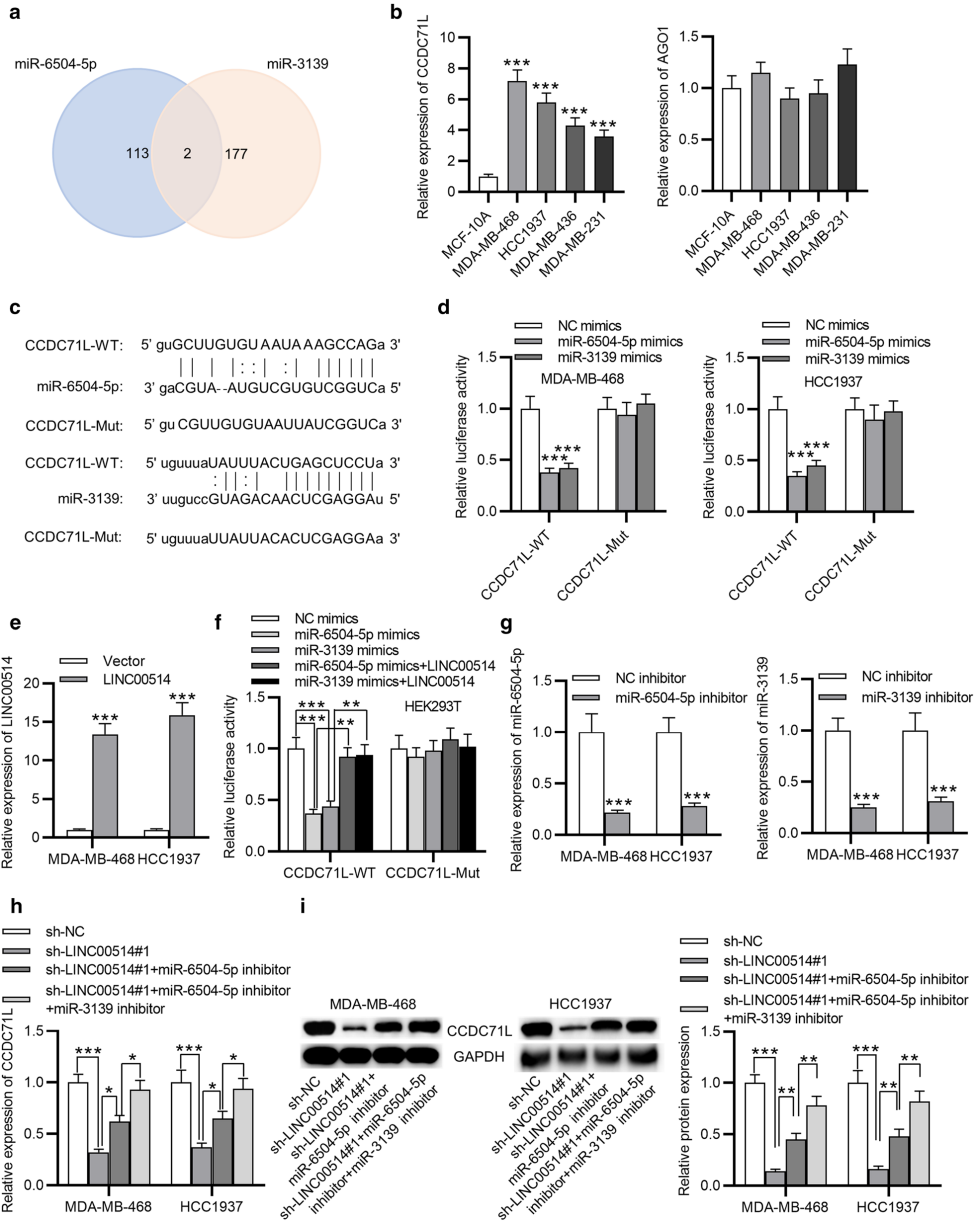
CCDC71L was targeted by miR-6504-5p and miR-3139. **a** Predicted targets for both miR-6504-5p and miR-3139 via starBase. **b** Expressions of CCDC71L and AGO1 in TNBC cells was analyzed by RT-qPCR. **c** The predicted binding sites and constructed mutant sites between CCDC71L and miR-6504-5p/miR-3139. **d** Luciferase activity of CCDC71L-WT/Mut in TNBC cells transfected with miR-6504-5p/miR-3139 mimics. **e** LINC00514 expression in TNBC cells with pcDNA3.1/LINC00514 transfection. **f** YAP1-WT/Mut luciferase activity with indicated transfection in HEK293T cell was verified by luciferase reporter assay. **g** Transfection efficiency of miR-6504-5p/miR-3139 inhibitor in TNBC cells was evaluated by RT-qPCR. (H-I) Detection of CCDC71L mRNA and protein levels in TNBC cells with transfection of indicated plasmids. *p < 0.05, **p < 0.01, ***p < 0.001

To further support ceRNA hypothesis, the downstream genes of miR-6504-5p and miR-3139 were explored. By using starBase, two potential mRNAs were found (Fig. 5a). Then, the expression of these two genes in TNBC cells were testified. As observed, CCDC71L was highly expressed in TNBC cells, while that of AGO1 did not exhibit expression difference (Fig. 5b). Subsequently, we found the binding site between CCDC71L and miR-6504-5p or miR-3139 to construct CCDC71L-WT, and mutated the sites to construct CCDC71L-Mut (Fig. 5c). After miR-6504-5p mimics or miR-3139 mimics transfection, the luciferase activity of CCDC71L-WT reporter was considerably decreased, while that of CCDC71L-Mut reporter remained unchanged (Fig. 5d). Additionally, LINC00514 was verified to be overexpressed in TNBC cells by pcDNA3.1/LINC00514 (Fig. 5e). Characterized by easy transfection, HEK293T cells were used for luciferase reporter assay. Data indicated that overexpressed LINC00514 counteracted the decrease in luciferase activity of CCDC71L-WT induced by overexpressed miR-6504-5p or miR-3139 while CCDC71L-Mut luciferase activity was not affected (Fig. 5f). Then, we confirmed that miR-6504-5p inhibitor and miR-3139 inhibitor apparently decreased miR-6504-5p and miR-3139 expression, separately, in TNBC cells (Fig. 5g). At last, we found that CCDC71L mRNA and protein levels decreased by silenced LINC00514 were partially restored by inhibiting miR-6504-5p, and the co-transfection of miR-6504-5p inhibitor and miR-3139 inhibitor nearly fully reserved the function of LINC00514 knockdown (Fig. 5h, i). Overall, LINC00514 increased CCDC71L expression via sponging miR-6504-5p and miR-3139 in TNBC.

### LINC00514 promoted TNBC cellular activities by upregulating CCDC71L or inhibiting miR-6504-5p and miR-3139

**Fig. 6:**
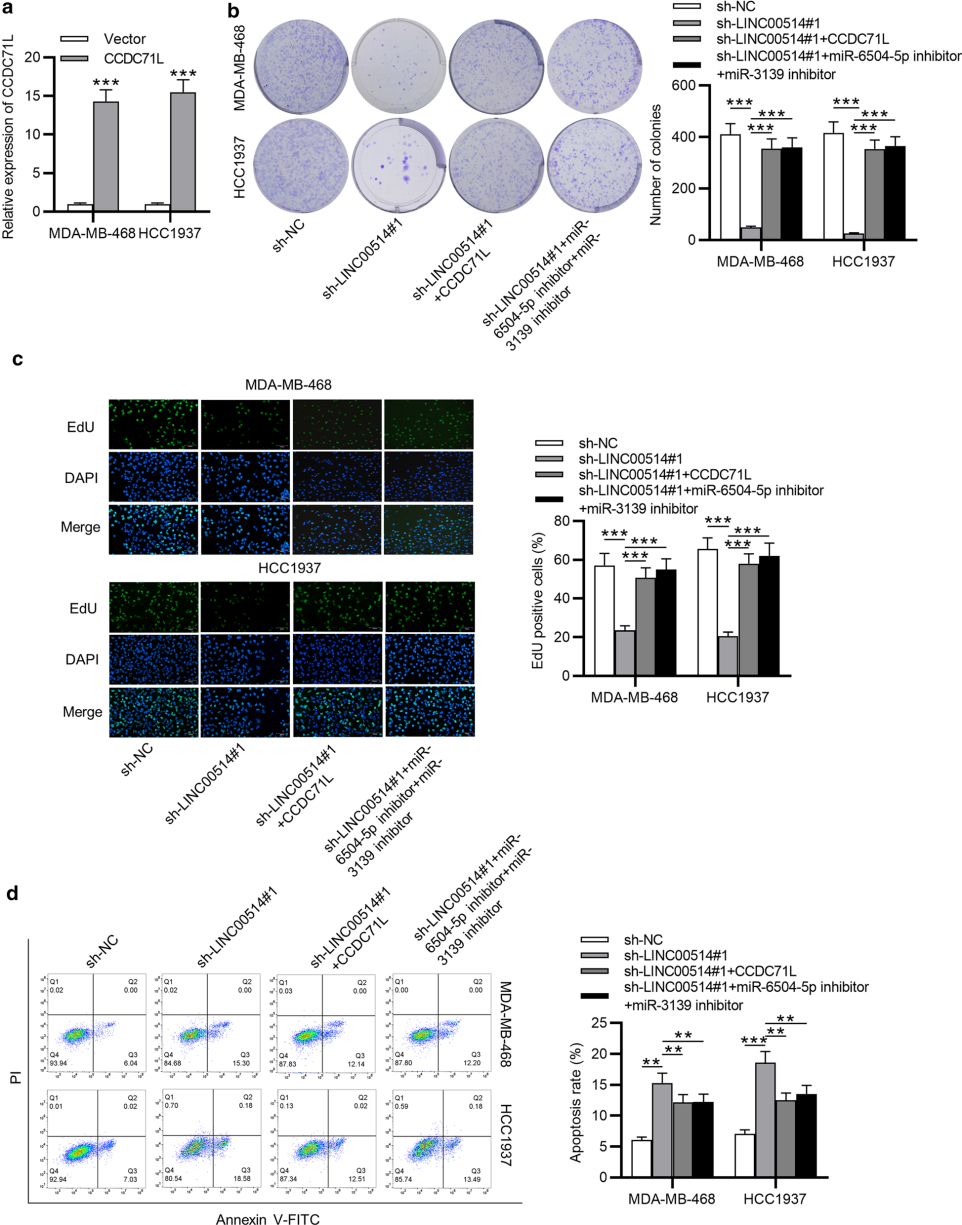

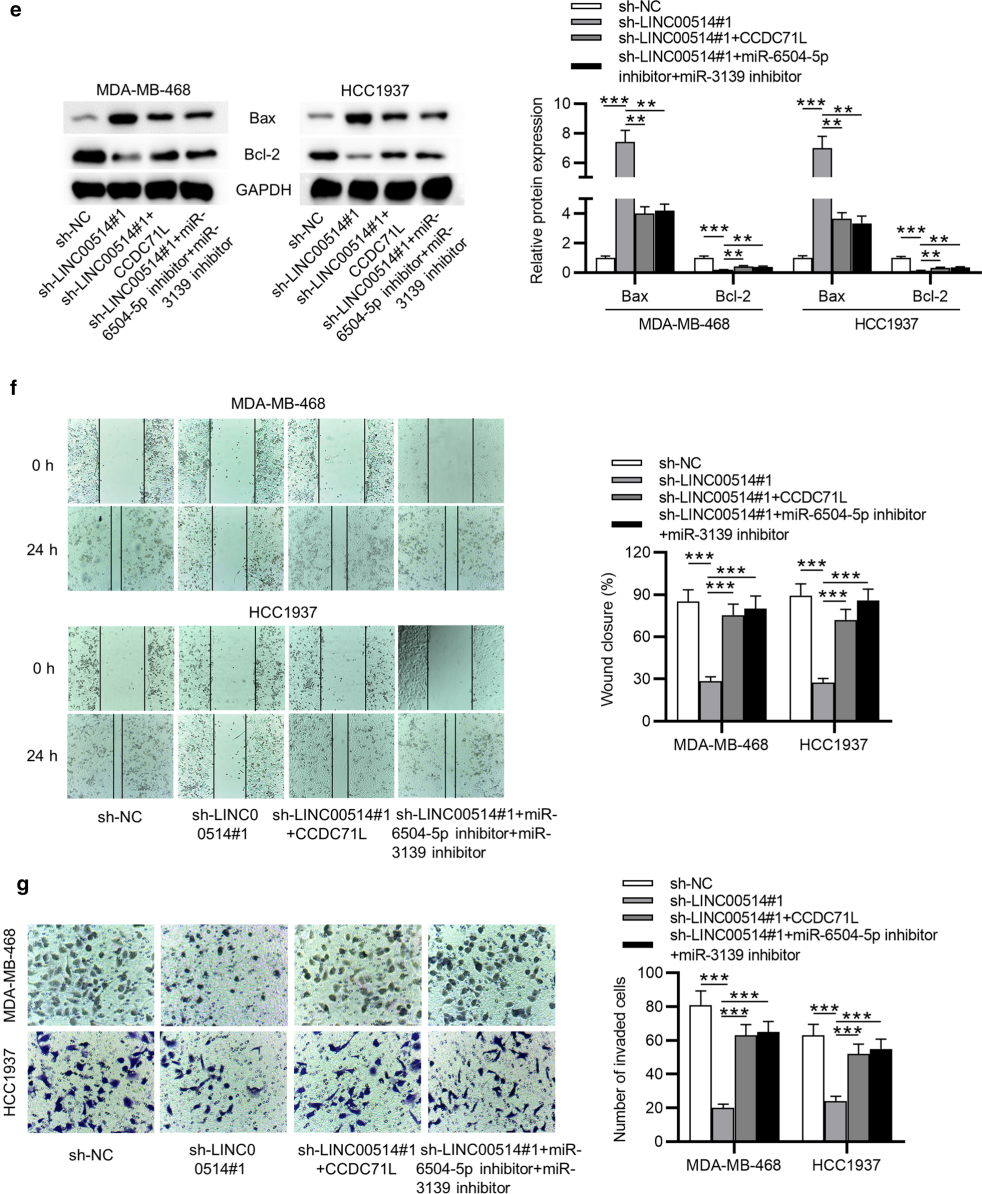
LINC00514 exerted regulatory functions on cellular activities via CCDC71L. **a** Transfection efficiency of pcDNA3.1/CCDC71L was confirmed. **b**, **c** Colony formation assay and EdU assay using MDA-MB-468 and HCC1937 cells with appointed transfection. **d**, **e** Cell apoptosis rate and proteins associated with apoptosis in each group were measured. **f**, **g** Cell migratory and invasive abilities with indicated transfection were assessed. *p < 0.05

For further analyzing whether LINC00514 played an oncogenic role in TNBC by mediating CCDC71L, some restoration experiments were conducted in MDA-MB-468 and HCC1937 cells. CCDC71L expression was upregulated in TNBC cells (Fig. 6a). As demonstrated, proliferative ability of TNBC cells inhibited by LINC00514 silencing was reversed via overexpressing CCDC71L or inhibition of miR-6504-5p and miR-3139 (Fig. 6b, c). Besides, the elevated cell apoptosis caused by LINC00514 knockdown was counteracted by CCDC71L overexpression or inhibition of miR-6504-5p and miR-3139 (Fig. 6d). Consistently, overexpressed CCDC71L or silenced miR-6504-5p and miR-3139 countervailed the effect of LINC00514 deficiency on apoptosis-related proteins levels (Fig. 6e). Meanwhile, LINC00514 knockdown-mediated inhibition on the migratory ability of TNBC cell was recovered by upregulating CCDC71L or silencing miR-6504-5p and miR-3139 (Fig. 6f). At last, upregulated CCDC71L or silenced miR-6504-5p and miR-3139 offset the suppressive role of sh-LINC00514 transfection in cell invasion (Fig. 6g). Hence, we validated that LINC00514 facilitated cell proliferation, suppressed cell apoptosis, and promoted cell migration and invasion via upregulating CCDC71L or downregulating miR-6504-5p and miR-3139 in TNBC.

## Discussion

In recent decades, extensive documents have strongly supported the participation of lncRNAs in the pathogenesis and development of human cancers [23–25]. The progression of TNBC was attributed to numerous oncogenes and anti-oncogenes [26, 27]. To promote TNBC therapy efficacies and develop novel TNBC treatments, it is critical to fully understand the molecular events, especially lncRNAs [14, 28]. Previous studies have demonstrated that LINC00514 accelerates cell proliferation and invasion in vitro and aggravates tumor growth by targeting the miR-204-3p/CDC23 axis in papillary thyroid cancer [19]. In osteosarcoma, LINC00514 increases URGCP expression to promote cell cycle and suppress cell apoptosis via sponging miR-708 [20]. However, the biological role of LINC00514 in TNBC remains unknown. Herein, this study was the first to reveal that LINC00514 exhibited a high level in TNBC tissues and cells, especially in the tissues of patients at advanced stage. Loss-of-function assay revealed that LINC00514 knockdown inhibited cell proliferation, induced cell apoptosis, suppressed cell migration and invasion, while in the human normal breast epithelial cell MCF-10 A, LINC00514 showed overexpression showed no evident influence on cell proliferation in TNBC. These findings suggested that LINC00514 served as an oncogenic lncRNA in TNBC.

Increasing reports indicated that lncRNAs serve as ceRNA to release miRNA targets by sequestering miRNAs [29]. To investigate the underlying regulatory mechanism of LINC00514-mediated cancer development, we conducted in silico studies to research putative miRNAs for LINC00514. Through bioinformatics analysis and RNA pull down assay, miR-6504-5p and miR-3139 were predicted to be potential miRNAs binding with LINC00514. It was supposed that miR-6504-5p and miR-3139 have the binding sites on LINC00514 sequences. Further, luciferase reporter assay confirmed the binding of LINC00514 to miR-6504-5p and miR-3139. In addition, we found that miR-6504-5p and miR-3139 were both lowly expressed in TNBC tissues and cells and their expression levels were negatively associated with that of LINC00514 in TNBC tissues. Therefore, we supposed that LINC00514 interacted with miR-6504-5p and miR-3139 in TNBC.

As another class of noncoding RNA, miRNAs only contain 20–24 nucleotides and exert critical effects on tumorigenesis and progression [30, 31]. MiR-6504-5p and miR-3139 are novel miRNAs whose functions and mechanism have not been depicted in cancers, especially in TNBC. In this study, miR-6504-5p and miR-3139 were both found to be downregulated in TNBC cell lines, and overexpression of miR-6504-5p or miR-3139 significantly inhibited cell proliferation, migration, invasion and contributed to cell apoptosis in TNBC. This confirmed the anti-oncogenic property of miR-6504-5p and miR-3139 in TNBC. Previously, increasing studies validated that miRNAs can directly bind to mRNA 3′-UTR to post-transcriptionally regulate mRNA translation or degradation [32]. Our present study identified CCDC71L, a novel mRNA that has not been explored in cancers, as the target of miR-6504-5p and miR-3139. CCDC71L was revealed to be highly expressed in TNBC cell lines. The binding between CCDC71L and miR-6504-5p and miR-3139 was verified by the luciferase reporter assay. MiR-6504-5p and miR-3139 target the 3’UTR of CCDC71L to suppress its expression. Importantly, we discovered that the inhibitive effect of silenced LINC00514 on CCDC71L mRNA and protein levels was partially restored by miR-6504-5p inhibition, and fully rescued by co-inhibition of miR-6504-5p and miR-3139. Rescue assays demonstrated that CCDC71L overexpression or miR-6504-5p and miR-3139 co-inhibition counteracted LINC00514 silencing-mediated suppression on TNBC cell proliferation, migration and invasion. Therefore, it was suggested that LINC00514 regulate CCDC71L expression via sponging miR-6504-5p and miR-3139.

LINC00514 is of clinical value as a promising therapeutic target of TNBC. The synthesized sh-LINC00514 provides a treatment option for TNBC patients at advanced stage. However, the relationship between LINC00514 and the prognosis in TNBC patients remains unclear. In the future, more studies need to be conducted to explore the association between the level of LINC00514 and TNBC prognosis.

In conclusion, our study for the first time revealed the functional role and molecular mechanism of LINC00514 in TNCB, and discovered that LINC00514 acted as a molecular sponge of miR-6504-5p and miR-3139 to increase CCDC71L expression, thereby promoting TNBC cell proliferation, migration and invasion (Additional file 2: Fig. S2). This finding might be helpful for the further exploration of new TNBC therapy.

## Supplementary Information

**Additional file 1: Figure S1.** (A) A colony formation assay was conducted to investigate the MCF10A cell proliferation after the transfection of pcDNA3.1/ LINC00514.

**Additional file 2: Figure S2. **LINC0051 promotes TNBC cell proliferation, migration, invasion and inhibits cell apoptosis by binding with miR-6504-5p and miR-3139 to upregulate CCDC71L at the posttranscriptional level.

## Data Availability

The datasets used during the current study are available from the corresponding author on reasonable request.
