# Correction to: LINC00514 upregulates CCDC71L to promote cell proliferation, migration and invasion in triple‐negative breast cancer by sponging miR-6504-5p and miR-3139

**DOI:** 10.1186/s12935-021-02262-7

**Published:** 2021-10-26

**Authors:** Xiao Luo, Hui Wang

**Affiliations:** 1 Department of Breast Surgery, China-Japan Union Hospital of Jilin University, Changchun, 130033 Jilin China; 2 Department of Ultrasound, China-Japan Union Hospital of Jilin University, Changchun, 130033 Jilin China

## Correction to: Cancer Cell Int (2021) 21:180 https://doi.org/10.1186/s12935-021-01875-2

Following the publication of the original article [1], we were notified of an error in Fig. 1B (Fig. 1). The previous sample sizes of clinical stage I–II (N = 52) and III–IV (N = 52) were mistakenly presented. It should be clinical stage I–II (N = 27) and III–IV (N = 25).

**Fig. 1:**
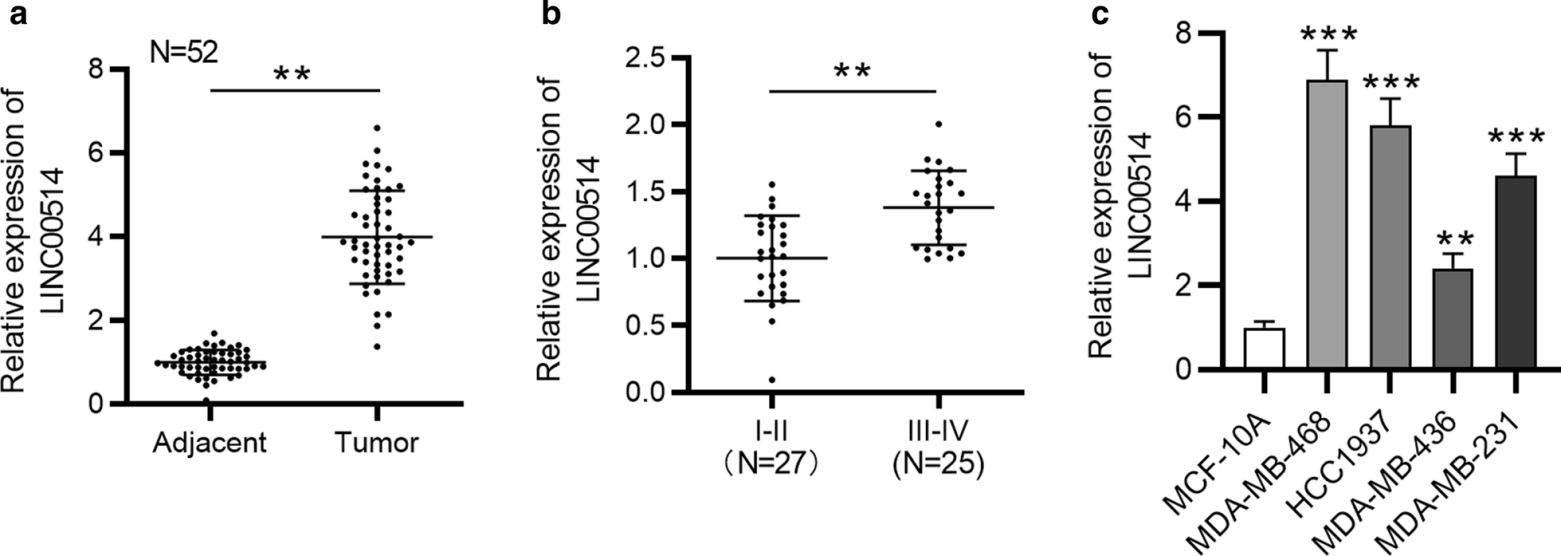
LINC00514 expression in TNBC tissues and cells. **a** RT-qPCR analysis of LINC00514 expression in TNBC tissues, with adjacent nontumor tissues as control. **b** Correlation of LINC00514 expression with clinical stage. **c** LINC00514 expression in TNBC cell lines was confirmed by RT-qPCR. **p < 0.01, ***p < 0.001

The correction does not change the interpretation or the original conclusions of this work. Therefore, the authors sincerely apologize for the oversight on this matter to the editors, reviewers and readers for any confusion that has been caused by this unintentional error.
