# Supplementary figures and images for: LINC00514 upregulates CCDC71L to promote cell proliferation, migration and invasion in triple‐negative breast cancer by sponging miR-6504-5p and miR-3139
Source: Cancer Cell Int. 2021 Mar 23;21:180. doi: 10.1186/s12935-021-01875-2 (PMC7986463; doi:10.1186/s12935-021-01875-2)

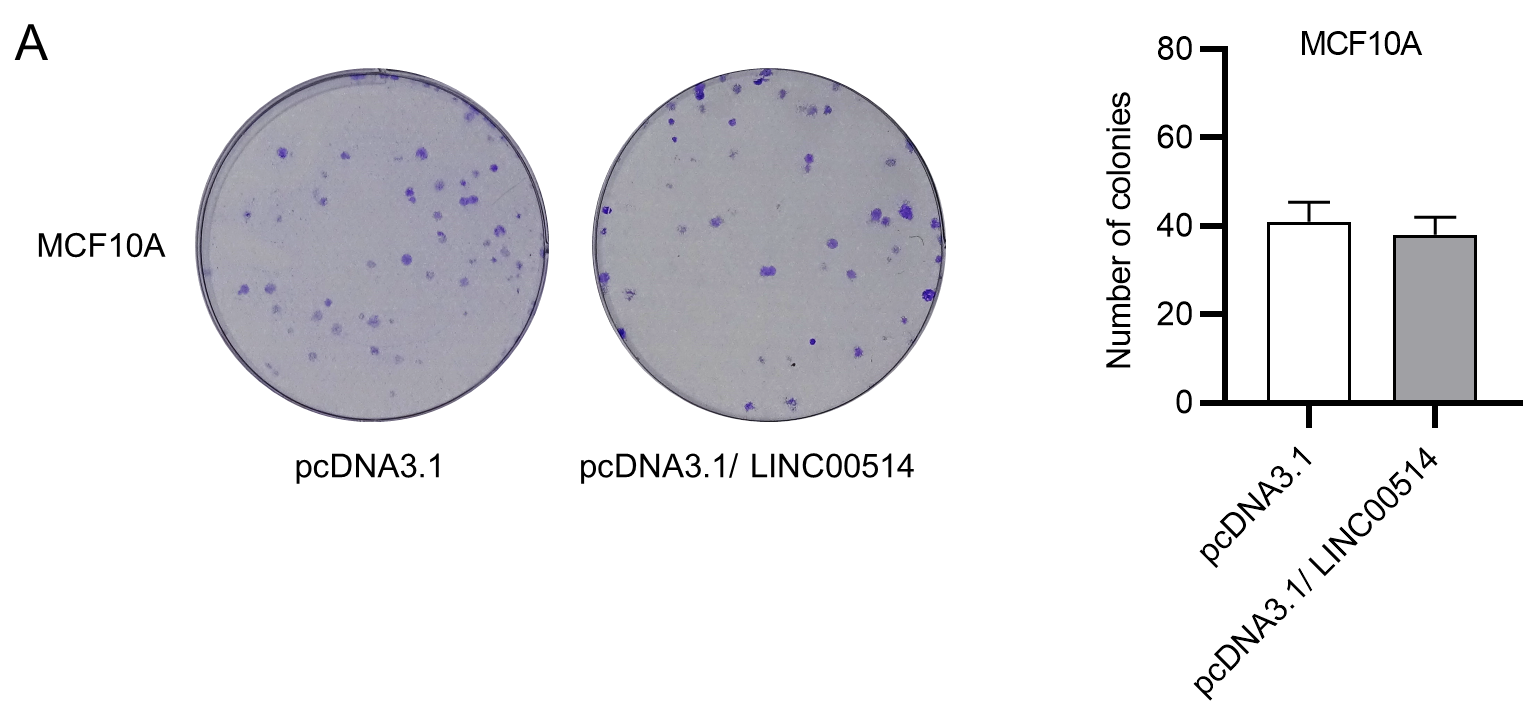

Supplement: Supplementary file 1 — Additional file 1: Figure S1. (A) A colony formation assay was conducted to investigate the MCF10A cell proliferation after the transfection of pcDNA3.1/ LINC00514. [file 12935_2021_1875_MOESM1_ESM.tif]

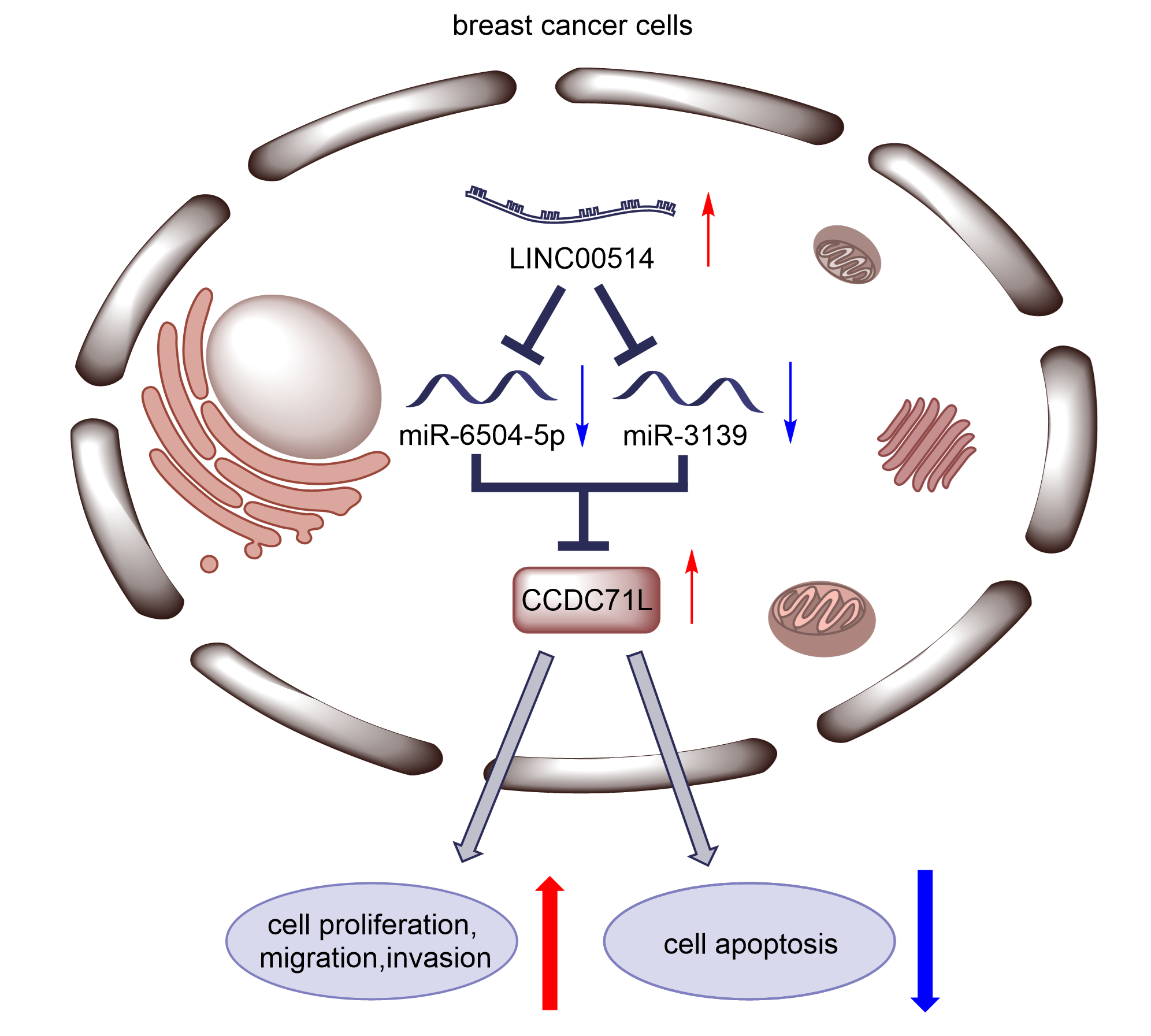

Supplement: Supplementary file 2 — Additional file 2: Figure S2. LINC0051 promotes TNBC cell proliferation, migration, invasion and inhibits cell apoptosis by binding with miR-6504-5p and miR-3139 to upregulate CCDC71L at the posttranscriptional level. [file 12935_2021_1875_MOESM2_ESM.tif]
